# Intraoperative Hemodynamic and Metabolic Stress as Determinants of Prolonged Intensive Care Unit Stay in Pediatric Liver Transplantation

**DOI:** 10.5152/eurasianjmed.2026.261516

**Published:** 2026-09-22

**Authors:** Taylan Şahin, Ali Sait Kavaklı, Alaaddin Aydın, Cansu Altuntaş, Mey Talip, Selma Kahyaoğlu, Neşe Kutlutürk Şahin, Ayhan Dinçkan

**Affiliations:** 1 Department of Anesthesiology and Reanimation, İstinye University Faculty of Medicine, İstanbul, Türkiye; 2 Department of Transplant Surgery, İstinye University Faculty of Medicine, İstanbul, Türkiye; 3 Department of Pediatric Gastroenterology, İstinye University Faculty of Medicine, İstanbul, Türkiye; 4 Department of Pediatric Intensive Care, İstinye University Faculty of Medicine, İstanbul, Türkiye; 5 Department of Anesthesiology and Reanimation, Bahçeşehir University Faculty of Medicine, İstanbul, Türkiye; 6 Department of Radiology, İstanbul Physical Therapy and Rehabilitation Training and Research Hospital, İstanbul, Türkiye

**Keywords:** Graft-to-recipient weight ratio, hemodynamic instability, intensive care unit, intraoperative lactate, pediatric liver transplantation, perioperative outcomes

## Abstract

**Background:**

This study aimed to identify perioperative determinants of prolonged intensive care unit (ICU) stay in a consecutive cohort of pediatric liver transplant recipients, with emphasis on intraoperative hemodynamic and metabolic variables.

**Methods:**

This retrospective, single-center cohort study included 112 consecutive pediatric patients who underwent orthotopic liver transplantation (LT). The primary outcome was ICU length of stay ≥7 days. A multivariable binary logistic regression model was constructed using preoperative and intraoperative variables exclusively, following formal multicollinearity assessment with cluster-based variable selection. Discriminative performance was assessed by receiver operating characteristic analysis with bootstrap-derived CIs.

**Results:**

Thirty-two patients (28.6%) experienced prolonged ICU stay. In a model limited by a low events-per-variable ratio (EPV 4.0), 2 variables were associated with prolonged ICU stay: higher graft-to-recipient weight ratio (GRWR; odds ratio [OR] = 1.840, 95% CI: 1.087-3.114; *P* = .023) and lower preoperative hemoglobin (OR = 0.715, 95% CI 0.521-0.981; *P* = .038). Peak intraoperative lactate demonstrated moderate standalone discriminative performance (area under the curve 0.745; optimal threshold 6.0 mmol/L, sensitivity 59.4%, specificity 88.7%), without retaining significance in the multivariable model owing to collinearity. Prolonged ICU stay was associated with significantly higher rates of reintubation (40.6% vs. 9.0%; *P* < .001) and 30-day mortality (31.2% vs. 8.9%; *P* = .007).

**Conclusion:**

Prolonged ICU stay in pediatric LT was associated with higher GRWR and lower preoperative hemoglobin. Peak intraoperative lactate showed moderate discriminative performance and a strong continuous association with ICU duration. These findings highlight perioperative correlates of ICU utilization that may inform risk stratification and warrant prospective evaluation.

**Main Points**

- In pediatric liver transplant recipients, higher graft-to-recipient weight ratio and lower preoperative hemoglobin independently predicted prolonged intensive care unit (ICU) stay (≥7 days)—both identifiable before surgery and useful for pretransplant risk stratification.
- Peak intraoperative lactate showed moderate discriminative performance (area under the curve: 0.745; optimal cutoff 6.0 mmol/L) and a strong continuous association with ICU duration (Spearman *r* = 0.411), making it a practical bedside marker of intraoperative physiological stress.
- Patients with prolonged ICU stay had markedly worse outcomes: 30-day mortality was more than three-fold higher (31.2% vs. 8.9%), reintubation exceeded 40%, and rates of sepsis and postoperative bleeding were significantly elevated.
- Intraoperative variables (lactate, mean arterial pressure, vasopressor exposure, ischemia duration) were highly correlated with each other, reflecting a single underlying physiological stress response rather than independent predictors; this collinearity was formally managed through cluster-based variable selection prior to modelling.

## Introduction

Liver transplantation (LT) remains the only curative intervention for children with end-stage liver disease, encompassing a heterogeneous spectrum of cholestatic, metabolic, and acute hepatic conditions. Over the past 3 decades, advances in surgical technique, immunosuppression, and multidisciplinary perioperative care have substantially improved outcomes, with contemporary 1-year patient survival rates exceeding 90% at high-volume centers.^1^^,^^2^ Despite these gains, the immediate postoperative period continues to impose significant physiological demands, and a clinically meaningful proportion of pediatric recipients require prolonged intensive care unit (ICU) admission. Prolonged ICU stay has been consistently associated with higher rates of postoperative morbidity—including reintubation, infectious complications, and postoperative bleeding—as well as increased resource utilization and inferior short-term survival.^3^^,^^4^

Existing literature on perioperative outcomes in pediatric LT has predominantly examined static preoperative characteristics—including recipient age, body weight, disease severity scores, and graft-to-recipient weight ratio (GRWR)—or long-term composite endpoints such as graft and patient survival.^5^^,^^6^ The GRWR, reflecting the size mismatch between the donor graft and the pediatric recipient, is a recognized determinant of early hemodynamic tolerance and portal hemodynamics; high GRWR values in small recipients may predispose to abdominal compartment effects and impaired venous outflow, increasing perioperative physiological burden.^7^ Despite the clinical relevance of these structural variables, the specific contribution of intraoperative physiological events to early ICU utilization remains comparatively undercharacterized. Liver transplantation imposes exceptional hemodynamic and metabolic challenges across 3 surgically distinct phases—dissection, anhepatic, and neohepatic—each associated with specific patterns of cardiovascular instability, coagulopathy, and metabolic derangement.^8^

Intraoperative hypotension, defined by sustained reductions in mean arterial pressure (MAP), and vasopressor dependence are recognized markers of hemodynamic instability during major abdominal surgery and have been independently associated with postoperative organ dysfunction and adverse outcomes.^9^^,^^10^ Peak intraoperative lactate—an integrative biomarker of global tissue hypoperfusion and impaired aerobic metabolism—provides dynamic, real-time information on the adequacy of oxygen delivery throughout the procedure. During the anhepatic phase, hepatic lactate clearance is transiently absent, rendering lactate levels particularly susceptible to accumulation; neohepatic lactate concentrations exceeding 5 mmol/L have been associated with a 50% probability of postoperative complications in adult LT recipients.^11^ Critically, unlike fixed preoperative recipient characteristics, hemodynamic targets and metabolic indices are directly amenable to intraoperative anesthetic optimization, suggesting them as potential targets for intraoperative optimization strategies that may reduce ICU utilization, though prospective evidence is required.^9^^,^^12^

Early allograft dysfunction (EAD), defined by the Olthoff criteria as the presence of serum bilirubin ≥10 mg/dL or international normalized ratio ≥1.6 on postoperative day 7, or aminotransferase elevation >2000 IU/L within the first 7 days, occurs in 20%-44% of LT recipients and is independently associated with prolonged ICU admission, higher transfusion requirements, and inferior graft survival.^13^^,^^14^ Despite the established prognostic significance of EAD, its relationship with intraoperative physiological determinants has not been systematically examined in the pediatric LT population. Furthermore, the substantial multicollinearity inherent among intraoperative variables—including the near-perfect correlation between anhepatic phase duration and cold ischemia time—has rarely been formally addressed in existing outcome analyses, potentially distorting the apparent independent contributions of individual predictors.^15^

The aim of this study was to identify perioperative determinants of prolonged ICU stay (defined as ≥7 days) in a consecutive cohort of 112 pediatric LT recipients, with particular emphasis on intraoperative hemodynamic and metabolic variables. This study hypothesized that markers of intraoperative physiological stress—including peak lactate level, magnitude of MAP reduction, and vasopressor exposure—as well as GRWR and preoperative anemia would be independently associated with prolonged ICU stay after adjustment for recipient characteristics and graft parameters.

## Material and Methods

### Study Design and Patients

This was a retrospective, single-center cohort study conducted at a tertiary pediatric LT center. The study was approved by the Ethics Committee of İstinye University Faculty of Medicine Review Board with waiver of individual informed consent (approval no.: 2026/3; date: March 13, 2026) and reported in accordance with the Strengthening the Reporting of Observational Studies in Epidemiology guidelines. All consecutive patients aged younger than 18 years who underwent orthotopic LT between January 2018 and December 2025 were eligible. Patients were excluded if they had undergone prior retransplantation, had incomplete perioperative records precluding primary outcome ascertainment, or died in the operating room before ICU admission. A total of 112 patients were included in the final analysis. An AI language model (Claude, Anthropic, accessed 2025) was used to assist with grammar correction and sentence structure in the preparation of this manuscript. All scientific content, data analyses, interpretations, and conclusions were produced exclusively by the authors.

### Data Collection

Data were extracted from electronic medical records by 2 trained investigators using a standardized case report form and underwent secondary audit for completeness. Preoperative data included patient demographics (age, sex, body weight, height, and body mass index), graft characteristics (graft weight and GRWR [%]), and laboratory parameters (hemoglobin, platelet count, international normalized ratio [INR], aspartate aminotransferase, alanine aminotransferase, total bilirubin, and albumin). Intraoperative data—obtained from anesthetic charts and monitoring records—included surgery and anhepatic phase duration, cold ischemia time, hemodynamic parameters (baseline and minimum MAP, MAP drop percentage, MAP drop score), vasopressor exposure (maximum norepinephrine dose and duration), blood product transfusion, and peak arterial lactate from serial blood gas analyses. Postoperative data included duration of mechanical ventilation, ICU and hospital length of stay, postoperative day 7 (POD7) graft function markers, postoperative complications, and 30-day all-cause mortality.

### Outcomes

The primary outcome was prolonged ICU stay, defined as ICU length of stay ≥7 days. This threshold was selected based on prior literature associating ICU admission exceeding 1 week with postoperative morbidity and resource utilization in pediatric solid organ transplantation. Secondary outcomes included mechanical ventilation duration, hospital length of stay, postoperative complication rates, and 30-day mortality. Early allograft dysfunction was defined according to the Olthoff criteria: bilirubin ≥10 mg/dL or INR ≥1.6 on POD7, or aminotransferase >2000 IU/L within the first 7 days.

### Statistical Analysis

Distribution of continuous variables was assessed using the Shapiro–Wilk test. Normally distributed variables (preoperative hemoglobin, surgery duration, and intraoperative minimum MAP; all Shapiro–Wilk *P* > .05) are reported as mean ± SD and compared using the independent-samples *t*-test. Non-normally distributed variables are reported as median (interquartile range) and compared using the Mann–Whitney *U*-test. Categorical variables are reported as number (%) and compared using the chi-square or Fisher exact test, as appropriate. A 2-tailed *P* < .05 was considered statistically significant.

A multivariable binary logistic regression model was constructed to identify independent predictors of the primary outcome. The model was restricted to preoperative and intraoperative variables; postoperative complications were excluded as candidate predictors to avoid reverse causation. Prior to model construction, pairwise Spearman correlation coefficients were computed to assess multicollinearity. Severe collinearity (*r* > 0.70) was identified among anthropometric variables (age, weight, height, GRWR; *r* = 0.80-0.98), hemodynamic variables (MAP baseline, minimum, and drop; *r* = 0.70-0.86), and ischemia-metabolic variables (cold ischemia time, anhepatic phase duration, norepinephrine exposure, and peak lactate; *r* = 0.69-0.97). One representative variable was retained from each correlated cluster: GRWR, MAP minimum, and peak lactate, respectively. The final model included 8 variables in 112 patients with 32 events (events-per-variable ratio: 4.0). This ratio falls below the commonly recommended threshold of 10, which may increase the risk of coefficient instability and overfitting; findings should therefore be interpreted with appropriate caution. No internal or external validation was performed, which further limits the transportability of the model. Cold ischemia time was defined as the interval from aortic cross-clamp of the donor to removal of the graft from cold storage immediately prior to implantation. Results are reported as odds ratios (ORs) with 95% CIs.

Discriminative performance of peak lactate and GRWR was assessed by receiver operating characteristic analysis; optimal thresholds were determined using the Youden index, and 95% CIs for the area under the curve (AUC) were estimated by nonparametric bootstrap resampling (1000 iterations). A sensitivity analysis was performed using an alternative ICU stay threshold of >3 days (cohort median); this included both univariable comparisons and a multivariable logistic regression model using the same 8-variable specification as the primary analysis (Table 1). All analyses were performed using Python (version 3.12; Python Software Foundation, Wilmington, DE, USA) with SciPy (version 1.11) and scikit-learn (version 1.4) libraries.

## Results

### Study Population

A total of 112 consecutive pediatric liver transplant recipients were included. The cohort comprised 82 males and 30 females (73.2%), with a median age of 42 months (interquartile range: 7-115) and median body weight of 13.0 kg (interquartile range [IQR]: 7.1-29.8). The overall median ICU stay was 3 days (IQR: 2-7). Based on the prespecified threshold, 80 patients (71.4%) were allocated to the short ICU stay group (<7 days) and 32 patients (28.6%) to the prolonged ICU stay group (≥7 days). Overall 30-day mortality was 15.2% (17/112); 1 patient had incomplete 30-day follow-up data and was excluded from mortality analysis only, while remaining in all other analyses. Baseline and perioperative characteristics are summarized in Tables 2-4.

### Preoperative Characteristics

Patients with prolonged ICU stay were significantly younger (9 [6-18] vs. 57.5 [9-121] months; *P* < .001), had lower body weight (7.10 [6.50-8.05] vs. 14.90 [8.41-32.90] kg; *P* < .001), and had markedly higher GRWR (3.26% [2.71-3.65] vs. 1.93% [1.38-2.97]; *P* < .001). Preoperative hemoglobin was lower in the prolonged ICU group (8.67 ± 1.51 vs. 9.72 ± 2.03 g dL^−1^; *P* = .009), as was serum albumin (*P* = .048); total bilirubin was higher (15.13 [6.72-22.45] vs. 4.20 [0.80-16.02] mg dL^−1^; *P* = .016). Platelet count, INR, and alanine aminotransferase did not differ significantly between groups (Table 2).

### Intraoperative Variables

Anhepatic phase duration was longer in the prolonged ICU group (41.50 [33.75-45.75] vs. 35.00 [30.00-40.50] min; *P* = .011), as was cold ischemia time (55.00 [43.25-65.25] vs. 40.00 [36.50-55.00] min; *P* = .016). Intraoperative hemodynamic instability was consistently more pronounced: minimum MAP was lower (39.00 ± 8.26 vs. 48.35 ± 10.20 mmHg; *P* < .001), relative MAP reduction was greater (33.08% vs. 14.61%; *P* = .009), and vasopressor exposure was higher—maximum norepinephrine dose (0.06 [0.04-0.10] vs. 0.02 [0.01-0.06] µg kg^−1^ min^−1^; *P* < .001) and duration (120 [37.5-220] vs. 36.5 [29.5-90] min; *P* = .002). Peak intraoperative lactate was markedly elevated (6.20 [4.10-8.00] vs. 3.30 [2.80-5.00] mmol L^−1^; *P* < .001), with a positive continuous association with ICU length of stay (Spearman *r* = 0.411; *P* < .001; Figures 1 and 2). Early allograft dysfunction was more frequent in the prolonged ICU group (59.4% vs. 33.8%; *P* = .023; Table 3).

### Postoperative Outcomes

Prolonged ICU stay was associated with substantially longer mechanical ventilation (48.00 [24.00-72.00] vs. 0 [0-0] hours; *P* < .001) and worse early allograft function, with higher POD7 bilirubin (4.65 [2.09-8.05] vs. 1.75 [1.06-3.82] mg dL^−1^; *P* < .001) and INR (1.65 [1.39-2.36] vs. 1.34 [1.22-1.65]; *P* < .001). Rates of reintubation (40.6% vs. 9.0%; *P* < .001), sepsis or major infection (18.8% vs. 3.8%; *P* = .017), and postoperative bleeding requiring intervention (22.6% vs. 6.3%; *P* = .034) were all significantly higher in the prolonged ICU group. Thirty-day mortality was 31.2% versus 8.9% (*P* = .007; Table 4).

### Multivariable Logistic Regression Analysis

In the final 8-variable multivariable model (n = 112, 32 events; events-per-variable ratio = 4.0), 2 variables were independently associated with prolonged ICU stay: higher GRWR (OR 1.840, 95% CI 1.087-3.114; *P* = .023) and lower preoperative hemoglobin (OR 0.715, 95% CI 0.521-0.981; *P* = .038). The attenuation of peak lactate reflects high collinearity with MAP minimum (*r* = −0.77) and cold ischemia time (*r* = 0.73), rather than an absence of clinical relevance (Table 5; Figure 3).

### Discriminative Performance

Peak intraoperative lactate (AUC 0.745, 95% CI 0.635-0.847; Youden-optimal threshold 6.0 mmol L^−1^, sensitivity 59.4%, specificity 88.7%) and GRWR (AUC 0.735, 95% CI 0.630-0.832; optimal threshold 2.36%, sensitivity 87.5%, specificity 63.7%) demonstrated the strongest standalone discriminative performance, both in the range of moderate discriminative ability (Figure 4).

### Sensitivity Analysis

Sensitivity analysis using an alternative ICU stay threshold of >3 days (cohort median; ≤3 days: n = 59; >3 days: n = 53) yielded results consistent with the primary analysis. Patients with ICU stay >3 days were younger, smaller, had higher GRWR, lower preoperative hemoglobin, higher peak lactate, and lower minimum MAP (all *P* < .01), supporting the robustness of primary findings (Table 1). Multivariable logistic regression using the >3-day threshold (n = 110, events = 53, events-per-variable ratio = 6.6) identified GRWR (OR 1.853, 95% CI 1.163-2.953; *P* = .009) and peak intraoperative lactate (OR 1.907, 95% CI 1.266-2.874; *P* = .002) as independently associated with the alternative outcome, further supporting the robustness of the principal findings (Table 1).

## Discussion

In this retrospective cohort study of 112 consecutive pediatric liver transplant recipients, several perioperative determinants of prolonged ICU stay (≥7 days), which occurred in 28.6% of the cohort, were identified. Higher GRWR and lower preoperative hemoglobin were independently associated with prolonged ICU stay in multivariable analysis. Peak intraoperative lactate demonstrated moderate standalone discriminative performance and a continuous positive association with ICU length of stay. Prolonged ICU stay was accompanied by substantially higher rates of reintubation, sepsis, postoperative bleeding, and 30-day mortality.

The identification of GRWR as an independent predictor of prolonged ICU stay is both clinically meaningful and mechanistically coherent. Patients with prolonged ICU stay had a markedly higher median GRWR (3.26% vs. 1.93%), and each 1-percentage-point increment in GRWR was associated with an 84% increase in the odds of ICU stay ≥7 days. Disproportionately large grafts impose mechanical constraints on hepatic venous outflow, compress adjacent vascular structures, and may precipitate intra-abdominal hypertension, all of which impair hepatic perfusion and prolong the systemic inflammatory response after reperfusion.^5^ A nationwide analysis of 10 000 living donor LT cases confirmed that GRWR ≥5% in pediatric recipients was associated with inferior patient survival.^6^ In the pediatric context, GRWR reflects not only anatomical fit but also recipient developmental physiology—smaller, younger recipients carry lower physiological reserve and greater vulnerability to hemodynamic fluctuation.^7^

Lower preoperative hemoglobin emerged as the second independent predictor (OR 0.715 per 1 g dL^−1^ increment; *P* = .038). Preoperative anemia in this population reflects the convergence of chronic disease-related erythropoietic suppression, hypersplenism, and dilutional effects of portal hypertension.^12^ Even moderate anemia may limit oxygen delivery reserve during the hemodynamic stress of the anhepatic and reperfusion phases, amplifying intraoperative metabolic burden.^4^ Importantly, unlike GRWR—which is largely determined by donor-recipient anatomy—preoperative hemoglobin represents a potentially modifiable target. Correction of anemia prior to transplantation through iron supplementation, erythropoiesis-stimulating agents, or preoperative transfusion strategies may reduce postoperative recovery burden, though this hypothesis warrants prospective evaluation.

Peak intraoperative lactate was among the most strongly differentiated univariable parameters (6.20 vs. 3.30 mmol L^−1^; *P* < .001), with a continuous positive association with ICU length of stay (Spearman *r* = 0.411; *P* < .001). Lactate reflects global tissue hypoperfusion and the cumulative adequacy of oxygen delivery across all 3 surgical phases; during the anhepatic phase, its accumulation is particularly pronounced owing to the transient loss of hepatic clearance.^8^ A neohepatic lactate threshold of approximately 5 mmol L^−1^ has been associated with a 50% probability of postoperative complications in adult LT recipients,^11^ and the Youden-optimal cutoff of 6.0 mmol L^−1^ is broadly consistent with this literature. Although peak lactate did not retain independent significance in the multivariable model due to collinearity with MAP minimum (*r* = −0.77) and cold ischemia time (*r* = 0.73),^16^ its moderate standalone AUC, bedside availability, and continuous relationship with ICU duration support its clinical utility as a real-time index of perioperative metabolic burden.

Patients with prolonged ICU stay demonstrated a consistent pattern of intraoperative hemodynamic compromise: lower minimum MAP (39.00 ± 8.26 vs. 48.35 ± 10.20 mmHg; *P* < .001), greater MAP reduction (33.08% vs. 14.61%; *P* = .009), and substantially higher vasopressor requirements. Prior large cohort studies in adult LT have demonstrated that sustained MAP below 50 mmHg independently predicts 30-day mortality and graft failure,^17^^,^^18^ and intraoperative hypotension has been associated with postoperative acute kidney injury.^9^ In the pediatric context, the consequences of hypotension are amplified by limited vascular reserve and developmental immaturity of autoregulatory mechanisms.

Thirty-day mortality was 31.2% versus 8.9% (*P* = .007)—a more than 3-fold difference. Early allograft function was also significantly impaired, consistent with the established association between EAD and prolonged ICU admission, greater transfusion requirements, and inferior graft survival.^14^^,^^19^

To the authors’ knowledge, this is one of the few studies in pediatric LT to explicitly restrict the multivariable model to preoperative and intraoperative variables, thereby preserving the temporal integrity of the causal framework and eliminating reverse causation bias. The most comparable prior study—Siddiqui et al^16^ (*Paediatr Anaesth*, 2023)—identified age <12 months, red blood cell transfusion >40 mL kg^−1^, and major postoperative respiratory events as predictors of prolonged ICU stay, but included postoperative variables as candidate predictors, potentially inflating apparent discriminative performance. The present analysis avoids this methodological pitfall by design.

Pairwise Spearman correlations revealed near-perfect correlations between cold ischemia time and anhepatic phase duration (*r* = 0.97), as well as strong intercorrelations among vasopressor exposure, lactate, and ischemia duration (*r* = 0.69-0.87). Cluster-based variable selection represents an advance in analytical rigor over prior pediatric LT outcome studies.^10^ The granular intraoperative phenotyping available in the present dataset—serial MAP measurements, vasopressor dosing records, and peak arterial lactate—is rarely captured in administrative or registry-based analyses,^20^ enabling a more physiologically nuanced characterization of ICU outcome determinants.

Graft-to-recipient weight ratio and preoperative hemoglobin—both ascertainable before surgery—enable prospective identification of high-risk recipients at the time of listing, facilitating proactive ICU planning. Preoperative hemoglobin optimization through iron supplementation, erythropoiesis-stimulating agents, and nutritional support represents a tractable intervention that has not been formally tested in prospective pediatric LT trials. The strong univariable associations of peak lactate (AUC 0.745) and hemodynamic parameters with ICU outcome are hypothesis-generating; intraoperative goal-directed strategies—including early vasopressor initiation at predefined MAP thresholds and lactate-guided resuscitation—represent plausible candidates for prospective evaluation, though no causal inference can be drawn from the present observational data. Whether real-time lactate monitoring above 6.0 mmol L^−1^.

Several limitations warrant acknowledgment. The retrospective single-center design limits generalizability and introduces the risk of unmeasured confounding. The sample size of 112 patients with 32 events yields an events-per-variable ratio of 4.0—below the recommended threshold of 10—raising the possibility of overfitting and coefficient instability; accordingly, the identified variables should be regarded as candidate associations rather than validated independent predictors. The absence of internal validation (e.g., bootstrap or penalized regression) and external validation limits confidence in transportability of findings. Calibration analysis and decision-curve analysis were not performed; predictive performance claims should therefore remain modest. Several clinically relevant variables were not available for inclusion in the multivariable model, including underlying diagnosis, disease severity indices (Pediatric End-Stage Liver Disease (PELD)/Model for End-Stage Liver Disease (MELD) score), donor type, urgency status, and preoperative ventilatory or ICU support. Given that younger age, lower body weight, higher GRWR, and disease severity are likely interrelated in this population, residual confounding from these unmeasured variables cannot be excluded. Additionally, primary diagnosis was not included as a covariate owing to coding heterogeneity and limited sample size. Missing data for height (n = 15) and cold ischemia time (n = 3) represent minor imprecision. The associations reported herein should be interpreted as perioperative correlates of ICU utilization; causal inference is not warranted from this observational design.

In pediatric liver transplant recipients, prolonged ICU stay (≥7 days) was associated with higher GRWR and lower preoperative hemoglobin in multivariable analysis. Peak intraoperative lactate demonstrated moderate standalone discriminative ability and a strong continuous association with ICU duration, while hemodynamic instability parameters consistently differentiated groups in univariable analysis. These findings are limited by a low events-per-variable ratio and the absence of validation, and should be interpreted as hypothesis-generating associations rather than validated predictors. Multicenter validation is required to establish clinical utility and inform risk-stratified perioperative management protocols.

## Figures and Tables

**Figure 1.**
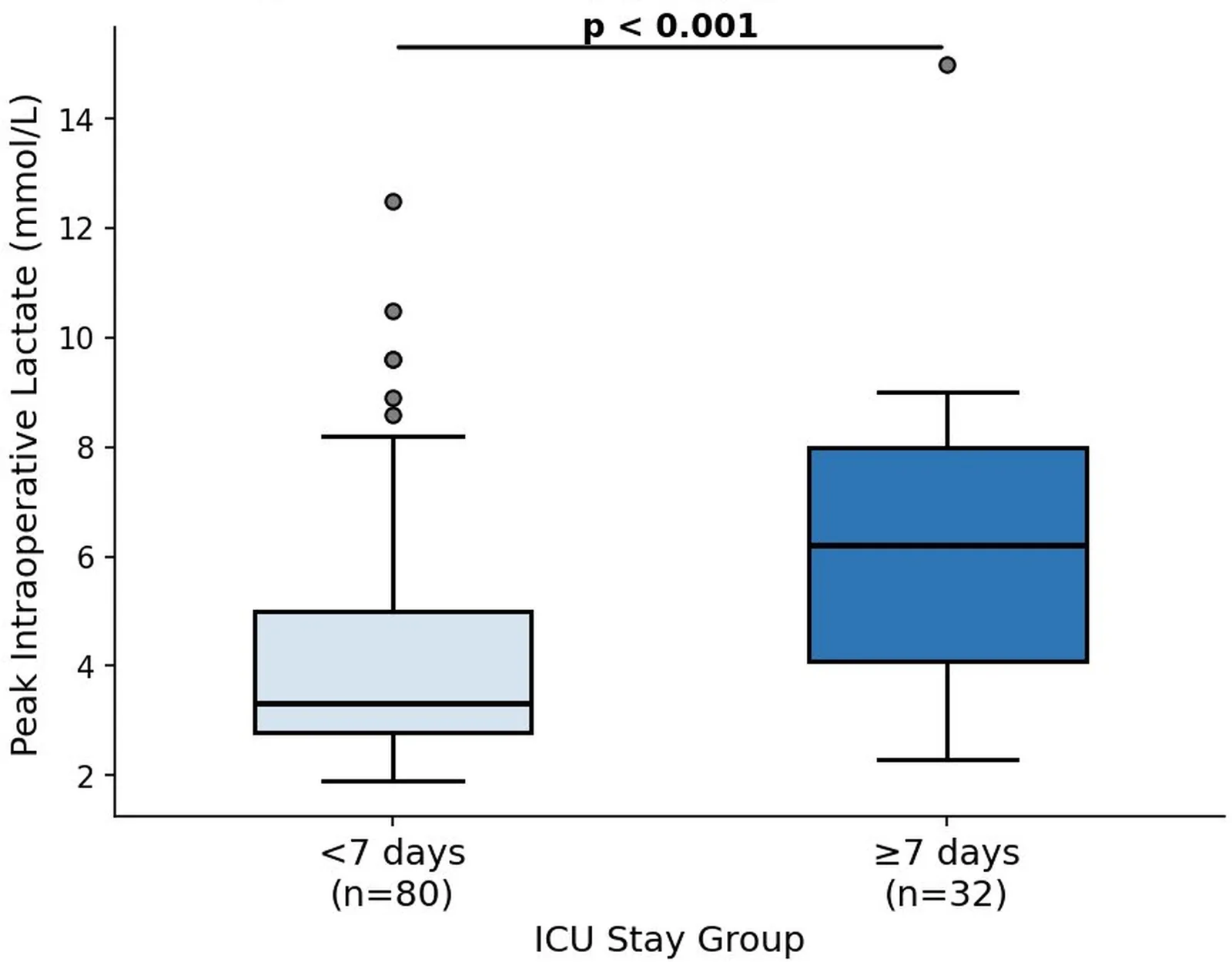
Distribution of peak intraoperative lactate levels (mmol L^−1^) in pediatric liver transplant recipients stratified by intensive care unit length of stay. The dashed line indicates the Youden-optimal threshold of 6.0 mmol L^−1^. Mann–Whitney *U*-test: *P* < .001.

**Figure 2.**
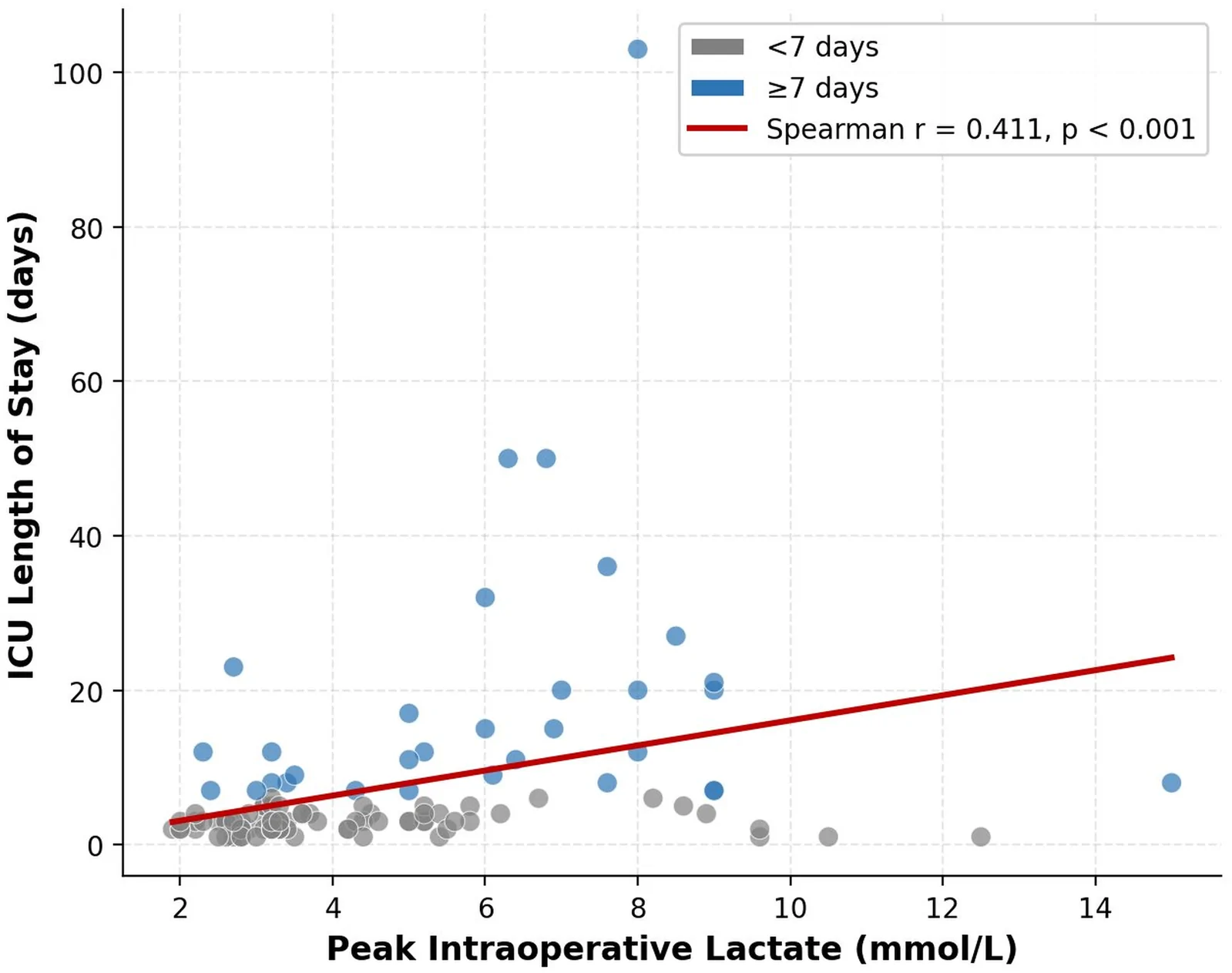
Scatter plot depicting the continuous relationship between peak intraoperative lactate (mmol L^−1^) and intensive care unit length of stay (days) across the full cohort. Spearman *r* = 0.411; *P* < .001.

**Figure 3.**
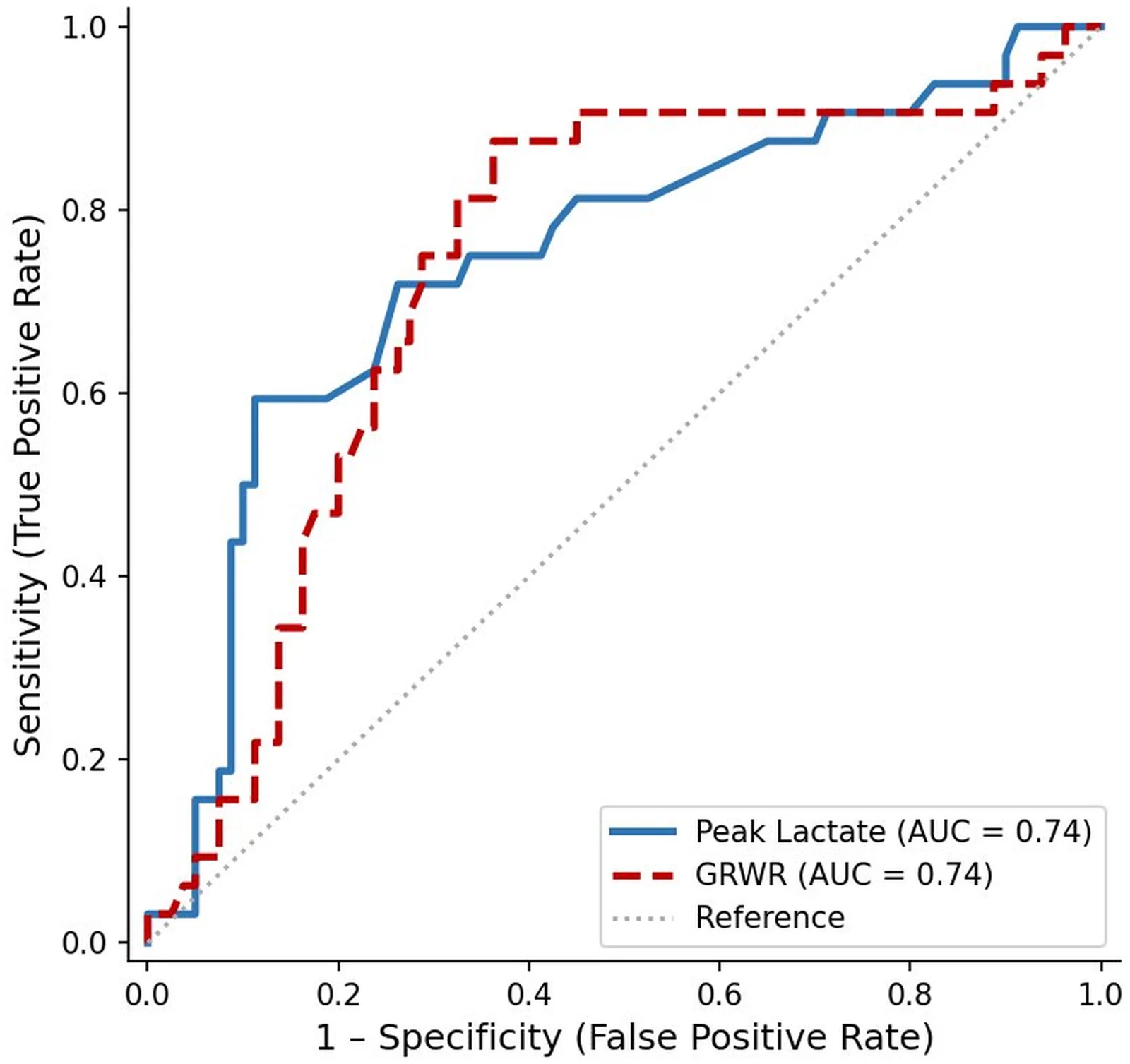
Forest plot of adjusted odds ratios with 95% CIs from the multivariable binary logistic regression model. GRWR, graft-to-recipient weight ratio; MAP, mean arterial pressure; OR, odds ratio.

**Figure 4.**
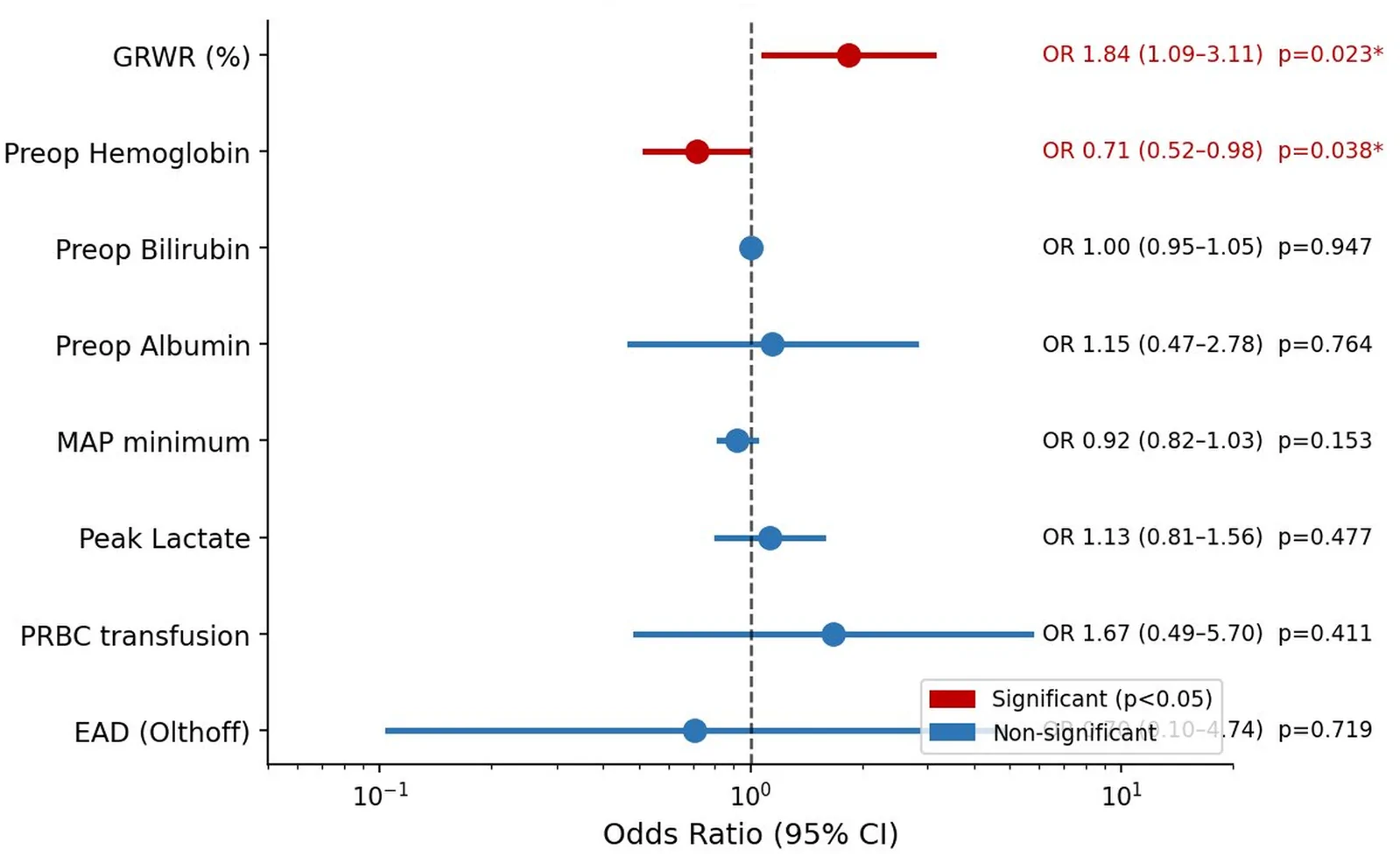
Receiver operating characteristic curves for peak intraoperative lactate (area under the curve 0.745, 95% CI: 0.635-0.847) and graft-to-recipient weight ratio (area under the curve 0.735, 95% CI: 0.630-0.832) as predictors of prolonged intensive care unit stay (≥7 days). Youden-optimal thresholds: lactate 6.0 mmol L^−1^; graft-to-recipient weight ratio 2.36%.

**Table 1.** Sensitivity Analysis—Alternative Intensive Care Unit Stay Definition (>3 Days)

Variable	≤3 days (n = 59)	>3 days (n = 53)	*P*
Age (months)	70.00 (12.00-143.50)	9.00 (6.00-20.00)	<.001
Body weight (kg)	18.00 (9.50-43.50)	7.45 (6.60-11.00)	<.001
Height (cm)	108.75 (75.25-150.25)	70.00 (65.00-87.00)	<.001
BMI (kg/m^2^)	16.27 (14.65-18.32)	15.19 (13.71-16.87)	.013
GRWR (%)	1.76 (1.36-2.56)	3.10 (2.19-3.65)	<.001
Preoperative hemoglobin (g/dL)*	9.86 ± 2.10	8.92 ± 1.64	.010
Preoperative bilirubin (mg/dL)	3.49 (0.64-13.20)	12.65 (3.20-23.20)	.006
Peak intraoperative lactate (mmol/L)	3.20 (2.75-4.35)	5.20 (3.40-7.60)	<.001
MAP minimum (mmHg)*	50.05 ± 10.00	40.81 ± 8.92	<.001
Reintubation, n (%)	5 (8.6)	15 (28.8)	.012
Sepsis/major infection, n (%)	1 (1.7)	8 (15.7)	.011
30-day mortality, n (%)	5 (8.6)	12 (22.6)	.074
**Variable **	**OR **	**95% CI **	***P***
**Panel A: Primary model—ICU stay ≥7 days (n = 110, events = 32, EPV = 4.0)**			
GRWR (%)	**1.944**	1.122-3.367	**.018**
Preoperative hemoglobin (g/dL)	0.751	0.533-1.059	.102
Preoperative bilirubin (mg/dL)	0.978	0.923-1.035	.435
Preoperative albumin (g/dL)	0.766	0.303-1.936	.573
MAP minimum (mmHg)	1.003	0.953-1.056	.903
Peak intraoperative lactate (mmol/L)	**2.063**	1.384-3.077	**<.001**
PRBC transfusion (units)	1.428	0.382-5.334	.596
EAD (Olthoff criteria)	2.167	0.518-9.072	.29
**Panel B: Sensitivity model—ICU stay >3 days (n = 110, events = 53, EPV = 6.6)**			
GRWR (%)	**1.853**	1.163-2.953	**.009**
Preoperative hemoglobin (g/dL)	0.864	0.665-1.124	.276
Preoperative bilirubin (mg/dL)	1	0.948-1.056	.991
Preoperative albumin (g/dL)	0.794	0.353-1.786	.577
MAP minimum (mmHg)	1.024	0.976-1.074	.333
Peak intraoperative lactate (mmol/L)	**1.907**	1.266-2.874	**.002**
PRBC transfusion (units)	1.153	0.414-3.212	.785
EAD (Olthoff criteria)	1.415	0.371-5.397	.612

Sensitivity analysis using the cohort-median ICU stay (3 days) as an alternative threshold. Results are broadly consistent with the primary analysis (≥7 days threshold), supporting the robustness of the principal findings. Continuous variables: median (IQR]), Mann–Whitney *U*-test. Sensitivity multivariable binary logistic regression analysis using alternative ICU stay threshold (>3 days).

Panel A reproduces the primary model (ICU ≥7 days) for comparison. Panel B presents the sensitivity model using the cohort median (3 days) as the alternative threshold. Both models include identical predictors following cluster-based collinearity adjustment. Bold values indicate *P* < .05.

BMI, body mass index; EAD, early allograft dysfunction (Olthoff criteria); EPV, events-per-variable ratio; GRWR, graft-to-recipient weight ratio; ICU, intensive care unit; MAP, mean arterial pressure; OR, odds ratio; PRBC, packed red blood cells.

*Normally distributed: mean ± SD, *t*-test. Categorical variables: chi-square or Fisher’s exact test as appropriate.

**Table 2.** Baseline and Preoperative Characteristics According to Intensive Care Unit Stay Group

Variable	<7 days (n = 80)	≥7 days (n = 32)	*P*
Demographics and anthropometrics			
Age (months)	57.50 (9.00-121.25)	9.00 [6.00-18.25]	<.001
Male sex, n (%)	60 (75.0)	22 (68.8)	.661
Body weight (kg)	14.90 (8.41-32.90)	7.10 (6.50-8.05)	<.001
Height (cm)	100.00 (72.00-143.00)	68.00 (64.00-80.00)	<.001
BMI (kg/m^2^)	16.07 (14.10-17.64)	15.40 (13.25-16.94)	.152
Graft characteristics			
Graft weight (g)	277.50 (230.00-388.75)	220.00 (200.00-271.25)	.003
GRWR (%)	1.93 (1.38-2.97)	3.26 (2.71-3.65)	<.001
Preoperative laboratory values			
Hemoglobin (g/dL)*	9.72 ± 2.03	8.67 ± 1.51	.009
Platelet count (×10^3^/µL)	146.0 (90.5-223.8)	199.5 (105.2-318.8)	.301
INR	1.31 (1.12-1.77)	1.44 (1.21-2.14)	.249
AST (U/L)	103.50 (48.75-304.75)	229.00 (106.25-372.00)	.049
ALT (U/L)	80.00 (31.75-154.25)	135.25 (52.75-248.25)	.067
Total bilirubin (mg/dL)	4.20 (0.80-16.02)	15.13 (6.72-22.45)	.016
Albumin (g/dL)	3.30 (2.77-3.90)	3.05 (2.68-3.40)	.048

Continuous variables are reported as median (IQR) unless normally distributed (Shapiro–Wilk *P* > .05).

ALT, alanine aminotransferase; AST, aspartate aminotransferase; BMI, body mass index; GRWR, graft-to-recipient weight ratio; ICU, intensive care unit; INR, international normalized ratio; IQR, interquartile range.

*Normally distributed: mean ± SD, compared using independent-samples *t*-test. All other continuous variables compared using the Mann–Whitney *U*-test. Categorical variables compared using the chi-square test.

**Table 3.** Intraoperative Variables According to Intensive Care Unit Stay Group

Variable	<7 days (n = 80)	≥7 days (n = 32)	*P*
Surgical parameters			
Surgery duration (min)*	358.06 ± 67.88	376.25 ± 79.18	.225
Anhepatic phase duration (min)	35.00 (30.00-40.50)	41.50 (33.75-45.75)	.011
Cold ischemia time (min)	40.00 (36.50-55.00)	55.00 (43.25-65.25)	.016
Crystalloid volume (mL)	500.00 (265.00-870.00)	310.00 (195.00-560.00)	.058
Hemodynamic parameters			
MAP baseline (mmHg)	62.00 (55.00-68.25)	55.00 (52.75-57.25)	<.001
MAP minimum (mmHg)*	48.35 ± 10.20	39.00 ± 8.26	<.001
MAP drop (%)	14.61 (10.16-36.40)	33.08 (13.12-42.43)	.009
MAP drop score	0.00 (0.00-2.00)	2.00 (0.00-2.00)	.010
Vasopressor and transfusion			
Norepinephrine max dose (µg/kg/min)	0.02 (0.01-0.06)	0.06 (0.04-0.10)	<.001
Norepinephrine duration (min)	36.50 (29.50-90.00)	120.00 (37.50-220.00)	.002
PRBC transfusion (units)	1.00 (0.00-1.00)	1.00 (1.00-1.00)	.025
FFP transfusion (units)	0.00 (0.00-0.00)	0.00 (0.00-1.00)	.355
Platelet transfusion (units)	0.00 (0.00-0.00)	0.00 (0.00-0.00)	.798
Metabolic parameters			
Peak intraoperative lactate (mmol/L)	3.30 (2.80-5.00)	6.20 (4.10-8.00)	<.001
EAD (Olthoff criteria), n (%)	27 (33.8)	19 (59.4)	.023

Continuous variables: median (IQR), Mann–Whitney *U*-test.

EAD, early allograft dysfunction; FFP, fresh frozen plasma; MAP, mean arterial pressure; PRBC, packed red blood cells.

*Normally distributed (Shapiro–Wilk *P* > .05): mean ± SD, *t*-test. Categorical variables: chi-square test.

**Table 4.** Postoperative Outcomes According to Intensive Care Unit Stay Group

Variable	<7 days (n = 80)	≥7 days (n = 32)	*P*
Clinical course			
Mechanical ventilation (h)	0.00 (0.00-0.00)	48.00 (24.00-72.00)	<.001
ICU length of stay (days)	3.00 (2.00-4.00)	12.00 (8.00-20.25)	<.001
Hospital length of stay (days)	13.00 (9.00-16.25)	13.50 (6.50-23.25)	.997
Graft function on POD7			
Bilirubin POD7 (mg/dL)	1.75 (1.06-3.82)	4.65 (2.09-8.05)	<.001
INR POD7	1.34 (1.22-1.65)	1.65 (1.39-2.36)	<.001
Peak AST POD7 (U/L)	47.00 (34.50-72.00)	44.50 (31.75-93.25)	.827
Peak ALT POD7 (U/L)	115.50 (74.25-171.25)	107.00 (57.25-277.50)	.486
Postoperative complications			
Major surgical complication, n (%)	12 (15.0)	9 (28.1)	.180
Reoperation/reintervention, n (%)	11 (13.9)	7 (21.9)	.456
Reintubation, n (%)	7 (9.0)	13 (40.6)	<.001
Sepsis/major infection, n (%)	3 (3.8)	6 (18.8)	.017
Postoperative bleeding requiring intervention, n (%)	5 (6.3)	7 (22.6)	.034
Allograft rejection, n (%)	17 (21.2)	7 (21.9)	1.000
30-day all-cause mortality, n (%)	7 (8.9)	10 (31.2)	.007

Continuous variables: median (IQR), Mann–Whitney *U*-test. Categorical variables: chi-square test; Fisher’s exact test for sepsis/major infection.

**Table 5.** Multivariable Logistic Regression Analysis for Prolonged Intensive Care Unit Stay (≥7 days)

Variable	OR	95% CI	*P*
Independent predictors (*P* < .05)			
GRWR (%)	1.840	1.087-3.114	.023
Preoperative hemoglobin (g/dL)	0.715	0.521-0.981	.038
Non-significant variables (*P* ≥ .05)			
Preoperative bilirubin (mg/dL)	1.002	0.953-1.053	.947
Preoperative albumin (g/dL)	1.146	0.472-2.784	.764
MAP minimum (mmHg)	0.921	0.822-1.031	.153
Peak intraoperative lactate (mmol/L)	1.126	0.812-1.561	.477
PRBC transfusion (units)	1.672	0.491-5.696	.411
EAD (Olthoff criteria)	0.705	0.105-4.735	.719

n = 112, events = 32, events-per-variable ratio = 4.0. The model was restricted to preoperative and intraoperative variables; postoperative complications were excluded to avoid reverse causation. Prior to model construction, pairwise Spearman correlation analysis was performed to address multicollinearity; one representative variable was retained from each correlated cluster (GRWR from the anthropometric cluster, MAP minimum from the hemodynamic cluster, peak lactate from the ischemia-metabolic cluster).

EAD, early allograft dysfunction (Olthoff criteria); GRWR, graft-to-recipient weight ratio; ICU, intensive care unit; MAP, mean arterial pressure; OR, odds ratio; PRBC, packed red blood cells.

## Data Availability

The data that support the findings of this study are available on request from the corresponding author.
